# Sex differences in contextual fear conditioning and extinction after acute and chronic nicotine treatment

**DOI:** 10.1186/s13293-024-00656-6

**Published:** 2024-10-31

**Authors:** Jack V. Keady, Marissa C. Hessing, Judy C. Songrady, Kristen McLaurin, Jill R. Turner

**Affiliations:** Department of Pharmaceutical Sciences, University of Kentucky College of Pharmacy, 789 S. Limestone Street, 473 Lee T. Todd Jr. Building, Lexington, KY 40536-0596 USA

**Keywords:** Hippocampus, Nicotine, Withdrawal, Fear conditioning, Extinction

## Abstract

**Background:**

Chronic cigarette smokers report withdrawal symptomology, including affective dysfunction and cognitive deficits. While there are studies demonstrating sex specific withdrawal symptomology in nicotine-dependent individuals, literature examining the underlying biological mediators of this is scant and not in complete agreement. Therefore, in this study, we evaluated the sex specific effects of nicotine and withdrawal on contextual fear memory, a hippocampally dependent aspect of cognition that is disrupted in nicotine withdrawal.

**Methods:**

Male and female B6/129F1 mice (8–13 weeks old) were used in all experiments. For the acute nicotine experiment, mice received intraperitoneal saline or nicotine (0.5 mg/kg) prior to contextual fear conditioning and test. For the chronic nicotine experiment, mice received nicotine (18 mg/kg/day) or saline for 11 days, then underwent contextual fear conditioning and test. Following the test, mice underwent minipump removal to elicit withdrawal or sham surgery, followed by the fear extinction assay. Bulk cortical tissue was used to determine nicotinic acetylcholine receptor levels via single point [^3^H]Epibatidine binding assay. Gene expression levels in the dorsal and ventral hippocampus were quantified via RT-PCR.

**Results:**

We found that female mice had a stronger expression of contextual fear memory than their male counterparts. Further, following acute nicotine treatment, male, but not female, subjects demonstrated augmented contextual fear memory expression. In contrast, no significant effects of chronic nicotine treatment on fear conditioning were observed in either sex. When examining extinction of fear learning, we observed that female mice withdrawn from nicotine displayed impaired extinction learning, but no effect was observed in males. Nicotine withdrawal caused similar suppression of *fosb*, *cfos*, and *bdnf*, our proxy for neuronal activation and plasticity changes, in the dorsal and ventral hippocampus of both sexes. Additionally, we found that ventral hippocampus *erbb4* expression, a gene implicated in smoking cessation outcomes, was elevated in both sexes following nicotine withdrawal.

**Conclusions:**

Despite the similar impacts of nicotine withdrawal on gene expression levels, *fosb*, *cfos*, *bdnf* and *erbb4* levels in the ventral hippocampus were predictive of delays in female extinction learning alone. This suggests sex specific dysfunction in hippocampal circuitry may contribute to female specific nicotine withdrawal induced deficits in extinction learning.

**Supplementary Information:**

The online version contains supplementary material available at 10.1186/s13293-024-00656-6.

## Plain text summary

Smokers undergoing nicotine withdrawal report increased feelings of anxiety, depression, and cognitive deficits. However, there are sex differences in these symptoms, with women reporting higher feelings of anxiety compared to men and men having worse cognitive deficits than women. The mechanisms underlying these sex differences in nicotine withdrawal symptoms are not well understood. The hippocampus is a brain region highly implicated in both the cognitive and anxiety-like symptoms of nicotine withdrawal. Therefore, we evaluated the effects of nicotine and withdrawal on contextual fear memory, a hippocampally dependent learning and memory task, in male and female mice. We found that female mice had a stronger contextual fear memory expression than their male counterparts. However, following acute nicotine treatment male mice had enhanced contextual fear memory compared to non-nicotine treated males, while acute nicotine had no impact on female mice. When examining extinction of contextual fear, we found female mice withdrawn from nicotine displayed impaired extinction learning, but no effect was observed in males. The female specific deficits in extinction learning due to nicotine withdrawal were correlated to hippocampal gene expression related to neuronal activity. This suggests hippocampal dysfunction may be driving the female specific nicotine withdrawal induced deficits in extinction learning.

**Supplementary Information:**

The online version contains supplementary material available at 10.1186/s13293-024-00656-6.

## Highlights

Female mice, but not males, showed deficits in contextual fear extinction during forced nicotine withdrawal.

Despite the sex specific impact of nicotine withdrawal on contextual extinction learning, with females alone showing deficits in extinction, we observed suppression of immediate early genes in the dorsal and ventral hippocampus and increased erbb4 mRNA in the ventral hippocampus in both sexes.

The deficits in female extinction were predicted by specific alterations in gene expression in the ventral hippocampus, highlighting a potential sex specific mechanism of nicotine withdrawal induced endophenotypes.

**Supplementary Information:**

The online version contains supplementary material available at 10.1186/s13293-024-00656-6.

## Introduction

Almost 70% of smokers in the United States express a desire to quit, and more than half of current smokers have made one quit attempt during the previous year [1]. Despite the desire and attempts to quit smoking, only 7.5% of smokers reported successful cessation during that same time frame [1]. The difficulties of maintaining smoking cessation in part can be attributed to the negative reinforcing effects of nicotine withdrawal [2–4]. The cessation of nicotine use results in cravings, affective dysfunction (e.g. increased feelings of anger, anxiety, and depression), and cognitive deficits (difficulty concentrating) [5–7]. Of these symptoms, difficulties concentrating or cognitive deficits due to nicotine withdrawal is a major predictor of relapse to smoking [8, 9]. Compounding these difficulties are the high rates of co-morbid psychiatric conditions amongst smokers [10–12]. The non-craving nicotine withdrawal phenotypes, specifically affective dysfunction and cognitive deficits, overlap with symptomology experienced in many of those psychiatric conditions. For instance, individuals suffering from post-traumatic stress disorder (PTSD) experience both affective dysfunction and cognitive deficits [13–15]. Furthermore, people suffering from PTSD are twice as likely to smoke, and half as likely to quit, compared to smokers without any diagnosed mental illness [12, 16]. Given that the symptomologies of PTSD and nicotine withdrawal overlap, there may as well be common underlying mechanisms, such as altered hippocampal cholinergic signaling [17, 18].

In addition to co-morbid psychiatric conditions, biological sex has major influences on nicotine withdrawal phenotypes. In women, nicotine withdrawal severity is associated with relapse to smoking, but this is not true in men [19]. Biological sex influences the presence and intensity of withdrawal symptoms as well. Using the a 72-point version of the ‘*Profile of Mood States’* (POMS) questionnaire, which includes 5 negative affect scales of anger, anxiety, confusion, depression, and fatigue, women experience larger changes in their POMS anxiety score during cessation relative to men after 16 hours of abstinence [20], but men have greater cognitive-related impairments during nicotine withdrawal than women [21, 22].

While there are several critical brain regions related to nicotine withdrawal symptomology, the hippocampus is an especially attractive circuitry node due to its involvement in both cognitive and affective disruptions occurring during nicotine withdrawal. For example, hippocampal gray matter volume has been associated with smoking cessation outcomes [23]. Both human subjects and rodents display impairments in hippocampus specific cognitive domains (i.e., contextual and spatial memory [24]) during withdrawal from chronic nicotine use [25–32]. While smokers have been shown to have deficits in both contextual fear inhibition [25] and differential fear conditioning [26], these studies were not powered to look at sex differences. Historically females have been excluded from the majority of research on learning and memory, fear conditioning, and fear extinction, with less than 2% of published research on this topic through 2012 including females [33]. This represents a significant caveat of these studies, as both male and female smokers have anatomical differences in hippocampal subregions [34]. Further, affective responding in male and females’ smokers has been linked to differential BOLD signaling in the hippocampus when presented smoking cues [35], a factor that may contribute to women experiencing higher rates of anxiety during nicotine withdrawal [20].

Contextual fear conditioning and extinction is a hippocampal-dependent fear learning and memory task that allows for investigation of the effects of chronic nicotine and/or spontaneous withdrawal on cognitive dysfunction. Functional imaging studies in men and women revealed hippocampal activation during fear acquisition, which gradually decreased during extinction [36]. Furthermore, in rodent studies, lesions to the hippocampus demonstrate deficits in contextual fear conditioning [37, 38]. Nicotine has a well-accepted role in modulating hippocampal-dependent learning. For example, acute nicotine enhances hippocampal-dependent learning memory tasks [39–41], and hippocampal specific injections of nicotine are sufficient to elicit these effects [42]. This relationship is more nuanced, however, as the hippocampus is not a homogenous structure with uniform function. Instead, dorsal and ventral subregions of the hippocampus underpin the contextual and affective associations to episodic memory, respectively [43]. Additionally, this distinct hippocampal spatial organization also displays unique transcriptional profiles [44], as well as distinct efferent and afferent projections [45–47]. This subdivision extends to nicotine’s effects as well. Direct injection of nicotine into the dorsal hippocampus (Dhip) before contextual fear conditioning increases percent time frozen in the contextual test, exposure to conditioning context with no shock presentation, while injections into the ventral hippocampus (Vhip) before conditioning caused deficits in fear learning in the contextual test [48], highlighting acute nicotine’s regiospecific impact on hippocampally dependent learning and memory. Furthermore, withdrawal from chronic nicotine causes deficits in contextual fear conditioning and extinction in male mice. However, female mice were not examined [27–29]. The literature on sex differences in nicotine withdrawal’s impact on hippocampally dependent learning and memory is not well established. Previous rodent studies found that both chronic nicotine exposure and spontaneous withdrawal inhibit contextual fear extinction in male mice, but chronic nicotine in females had no impact, and nicotine withdrawal was not investigated in females [29, 49].

Building upon this, we examined the effects of sex on responses to acute nicotine, chronic nicotine, and nicotine withdrawal in a contextual fear conditioning and extinction paradigm. In addition to these endpoints, we also evaluated transcriptional changes in genes linked to neuronal activation and plasticity (*fosb*, *cfos*, *bdnf*) [50–52], genes implicated in PTSD, a psychiatric disorder heavily co-morbid with nicotine use disorder (*fkbp5*, *brd4*, *crf*) [12, 53–57], as well as smoking cessation outcome linked genes (*nrg3* and *erbb4*) [58, 59].

## Methods

### Animals

Male and female B6/129F1 mice (8–13 weeks of age; 17–33 g; Taconic) were housed in groups of 3–4 same sex mice per cage. Estrus cycle was not tracked in females based on previous expert reviews showing variability due to cycle is negligible [60, 61]. The mice undergoing chronic treatment were 8–13 weeks of age at the beginning of saline or nicotine treatment. Mice were maintained on a 12-hour light-dark cycle (lights on at 7:00 AM), with ad libitum food and water in accordance with the University of Kentucky’s Institutional Animal Care and Use Committee. All experiments were conducted between 0800 and 1500. Animals were randomly assigned to treatment groups and were weight counterbalanced.

### Drugs and administration

For all experiments, (-)-Nicotine tartrate (MP Biomedicals, Solon, OH.) was dissolved in 0.9% saline and reported nicotine doses are in free base weight. For acute studies, nicotine was dissolved in saline and pH adjusted to neutrality with 0.1 M NaOH. For acute studies, nicotine was administered intraperitoneally (i.p.) at a dose of 0.5 mg/kg, as this dose has been shown previously to enhance fear conditioning in male mice [40] as well as male and female mice with no observed sex differences [39]. Nicotine or saline was administered i.p. 10 min prior to behavioral sessions. All mice were handled and received i.p. injections of saline for 5 days prior to start of acute nicotine studies to minimize the stress of injections on behavioral test days. All i.p. injections were administered at a volume of 0.01 ml/g.

For chronic studies, nicotine was administered subcutaneously via osmotic minipumps (Alzet model 1002, Cupertino, CA) at a dose of 18 mg/kg/day for 11 days. This dose is based on previous work [58, 62–64] and corresponds to plasma levels of ∼ 0.3 µM [65], a concentration similar to that observed in human smokers consuming an average of 17 cigarettes a day (plasma levels between 0.06 and 0.31 µM) [65]. Nicotine or saline was released intermittently via a coil attached to the osmotic minipumps [66]. A 24 inch of BTPE-60 tubing (Instech Laboratories, Plymouth Meeting, PA) was wrapped around a 0.1 ml syringe and placed into a beaker of boiling water for 2 min, followed by cooling with ice water. The resulting coils were alternatingly filled with either 0.25 µL of the nicotine solution or saline solution and mineral oil using two syringe pumps (Model GenieTouch, Kent Scientific, Torrington, CT) and a Y-connector (Instech Laboratories, Plymouth Meeting, PA). The 0.25 µL volume was chosen as this is the same volume dispensed by the osmotic minipump in a 1-hour time frame so that mice receive 1 h of nicotine exposure followed by 1 h of mineral oil (abstinence). Final nicotine concentrations using this methodology resulted in a dose of 18 mg/kg/day.

### Osmotic minipumps surgeries

In chronic nicotine studies, animals were implanted with coils fixed to osmotic minipumps to deliver either nicotine (18 mg/kg/day) or saline. Pump implantation was performed as previously described [67]. Briefly, mice were anesthetized with 1–3% isoflurane, and pumps attached to either nicotine or saline coils were implanted subcutaneously via a small incision on their right flank and closed with 7 mm stainless steel wound clips. Following 11 days of chronic administration, mice were anesthetized with an isoflurane/oxygen vapor mixture (1–3%), an incision was made above the pump at shoulder level and the pump was either removed (to initiate spontaneous withdrawal from either nicotine or saline) or left in place (to serve as sham surgical controls in the nicotine and saline groups). The incision was then closed with 7 mm stainless steel wound clips to induce spontaneous withdrawal.

### Fear conditioning and extinction

The contextual fear conditioning and extinction experiments were conducted using the Ugo-Basile Fear Conditioning Boxes and analyzed using Anymaze behavioral software provided by the University of Kentucky Rodent Behavior Core. The protocol for conditioning and extinction was modified from previous approaches [29, 49]. Briefly, mice were acclimated to the testing room for 1 h prior to the start of assessments. For conditioning sessions, mice were placed in the fear conditioning boxes for a 330s session. Baseline freezing was assessed during the first 120 s, followed by the onset of the first Conditional Stimulus (CS) (89dB tone) - Unconditional Stimulus (US) (foot shock) pairing. The 89dB tone (CS) was presented for 30s and paired with a light, and during the last 2 s of the CS presentation, the 2s foot shock (US) was presented. The CS and US then co-terminated 30 s after the CS was presented, followed by a 120s inter-trial interval. After the inter-trial interval, the second CS-US pairing was presented under the same conditions as the first, and the mice remained in the chamber for another 30 s. The foot shock intensity was either 0.35 mA or 0.5 mA depending on the experiment. The US shock intensity was the same for all mice in each cohort. For test and extinction sessions, mice were placed back into the same context for 300s without presentation of the CS or US. Freezing behavior was assessed for the entire extinction session (300s). Freezing behavior was defined as bouts of no movement except for breathing for longer than 1s, which was specified in the Anymaze software for video analysis. For all behavioral tests in the fear conditioning boxes, a background white noise of 68dB was played.

### [^3^H]epibatidine binding assay

Radioligand binding was performed as previously described [63]. Cortical tissues were homogenized in 50 mM Tris HCl buffer, pH 7.7 at 4 °C, and centrifuged twice for 15 min at 30,000 g in the cold centrifuge set to 4 °C in fresh buffer. The membrane pellets were resuspended in fresh buffer and added to tubes containing 1.5 nM [^3^H]Epibatidine ([^3^H]EB, PerkinElmer, Boston, MA, USA) with or without 300 µM nicotine, to determine specific binding, and incubated for 2 h at room temperature. Bound receptors were separated from free ligand by vacuum filtration over GF/C glass-fiber filters (Brandel, Gaithersburg, MD, USA) that were pre wet with 0.5% polyethyleneimine. The filters were then incubated in scintillation fluid overnight and counted in a liquid scintillation counter the following morning. Specific binding was defined as the difference between total binding, incubation with no nicotine, and nonspecific binding, incubation with 300 µM nicotine.

### Quantitative RT-PCR

Quantitative reverse transcriptase PCR was performed as previously described [68] on ventral and dorsal hippocampal samples across all treatment groups. Briefly, RNA was isolated using the RNeasy Mini kit (Qiagen) and complementary DNA was synthesized from 500 ng of the isolated RNA. Primers were designed using Primer3Plus, and qPCR reactions were 7 uL in volume assembled using cDNA, Thermo Scientific Maxima SYBR Green master mix, and 100nM primer (Integrated DNA Technologies, Coralville, IA). The mRNA levels of our genes of interest were determined using the 2^−ΔΔCT^ method [69] normalized to the housekeeping gene TATA-Binding Protein (*tbp*). All gene expression values were normalized to female saline treated controls within hippocampal subregions. Primer sequences are shown in Table 1.

**Table 1:** Sequence of primers used in qPCR

Gene	Forward Primer	Reverse Primer
*fosb*	GGTGAGGGCTATGGAGTCAA	GTCTCGCTCAGACCACTTCC
*cfos*	TGTCCGTCTCTAGTGCCAACT	CTGCTCTACTTTGCCCCTTCT
*bdnf*	AGGCAAACAATCGCTTCATCT	CAGGCTAACTCGAAAGGAACG
*fkbp5*	ACTGACTGACTGGCCTGCTAA	TCTACCCACTCTCCACACCAC
*brd4*	GTGCCTGGTGAAGAATGTGAT	GTTAGGGTTGGAGGTCTCTGG
*crf*	CTGGATCTCACCTTCCACCTT	TGTGTGCTAAATGCAGAATCG
*nrg3*	CAGCTGTGGTGTGTTGAAAGA	GGGGTTTGTCTCTCTTGAAGG
*erbb4*	ACAACCAGCACCATACCAGAG	TGTCATGCATTGGAGTCATGT
*tbp*	GCACAGGACTTACTCCACAGC	GTGGGTTGCTGAGATGTTGAT

Abbreviations: *fosb*, FosB Proto-Oncogene, AP-1 Transcription Factor Subunit; *cfos;* Fos Proto-Oncogene, AP-1 Transcription Factor Subunit; *bdnf*, Brain Derived Neurotrophic Factor; *fkbp5*, FK506-Binding Protein 5; Bromodomain Containing 4; *crf*, Corticotropin Releasing Hormone; *nrg3*, Neuregulin 3; *erbb4*, Erb-B2 Receptor Tyrosine Kinase 4; *tbp*, TATA-Box Binding Protein

All tissues were collected 2 h after the final test session. We choose 2 h given the wealth of data that this timepoint captures the peak of *cfos* expression as well as the induction of other target genes of interest (*fosb*, *bdnf*, etc.) [70].

### Experiment 1 methods: Acute Nicotine and Fear Conditioning

Separate cohorts of animals were used for the 0.5 mA acute nicotine fear conditioning experiment and the 0.35 mA acute nicotine fear conditioning experiment. For 5 days prior to the start of acute nicotine fear conditioning experiments, all mice were handled for 2 min a day, weighed, and given an i.p. injection of saline to try and minimize any acute stress of saline or drug treatment on conditioning and test days. On day 1 mice were acclimated to the behavior room 1 h prior to the start of contextual fear conditioning at 0830. Mice were treated with acute saline or nicotine i.p. 10 min prior to the start of the contextual fear conditioning session. Mice then underwent contextual fear conditioning with a US of 0.5 mA. After the mice completed conditioning, they were returned to their home cage. Following completion of conditioning for all animals, mice were returned to the colony room. On day 2, mice again were acclimated to the behavior room 1 h prior to the start of the contextual test at 0830. Mice were treated with acute saline or nicotine i.p. 10 min prior to the start of the test. Immediately after completion of the test, mice were placed back into their home cage and taken to a surgery suite for sacrifice, 5 to 10 min after completion of the test.

While we observed a robust effect of time, with conditioning causing an increase in percent time frozen in the test and an increase in percent time frozen in the acute nicotine group, the effects of acute nicotine did not reach statistical significance. Believing the statistical significant in the acute nicotine group was due to a ceiling effect and or potential sex differences, using naïve mice we decreased our shock intensity to 0.35 mA and increased our power to investigate sex (*N* = 6–8 sex*drug). All procedures in this experiment are identical to the pervious paragraph, except shock intensity was lowered to 0.35 mA.

### Experiment 2 methods: chronic nicotine and spontaneous withdrawal fear extinction

A new separate naïve cohort of mice was used for the chonric nicotine and withdrawal experiment. All mice were handled for 2 min a day and weighed for 4 days prior to minipump implantation surgery to familiarize the animals with the experimenter. On day 1, mice were implanted with osmotic minipumps with coils containing saline or nicotine. Mice were handled daily to assess recovery and health following the surgery. On day 12, mice were acclimated to the behavior room 1 h prior to the start of fear conditioning at 0830. Mice underwent contextual fear conditioning with a 0.50 mA US. Following completion of the conditioning mice were returned to their home cage. After all mice completed their conditioning sessions they were returned to the colony room. On day 13, mice were again acclimated to the behavior room 1 h prior to the start of the test. Following completion of the test mice were returned to their home cage, then returned to the colony room after all mice completed their sessions. Minipump removal and sham surgeries were performed in the afternoon of day 13, 3 h after the test. Following surgery and recovery mice were returned to the colony room. On days 14–17 mice were acclimated to the behavior room at 0830 for 1 h prior to extinction sessions, (i.e., E1-E4). Extinction sessions were conducted in the same context as conditioning, and no CS or US were presented during the 300s exposure period. Extinction sessions were the same for all 5 sessions. Following completion of the extinction session mice were returned to their home cage, then returned to the colony room after all mice completed their sessions. On day 18, mice were again acclimated to the behavior room 1 h prior to the start of the last extinction session (E5) at 0830. Mice were sacrificed 2 h after completion of the last extinction session and the hippocampi were microdissected into dorsal hippocampus and ventral hippocampus subregions to be used for RT-PCR analysis. Our lab has previously shown that CREB deletion in the Vhip increases percent time freezing in contextual fear conditioning, but CREB knockdown in the Dhip decreases percent time freezing [71], highlighting the unique subregional contributions of the hippocampus contextual memory. Therefore, we investigated the potential impacts of drug treatment on these subregions in the context of contextual fear extinction. Bulk cortical tissue was collected for ligand binding assay. All tissues were flash frozen on dry ice and stored at -80 °C until processing.

### Statistical analyses

In experiment 1, acute nicotine and fear conditioning, statistical analyses were performed with GraphPad Prism 10.0 software package (GraphPad Software, CA). The 0.5 mA fear conditioning results were analyzed using a two-way repeated measures ANOVA, treating time and drug treatment (referred to as drug from here on out) as factors, followed by a Sidak’s multiple comparison test (*N* = 8 per drug group). Time defined as the 24 h period after conditioning when mice are placed back into the conditioning context. The 0.35 mA fear conditioning was analyzed using three-way repeated measures ANOVA (*N* = 6–8 per sex*drug group), and time, drug, and sex were treated as factors, followed by a Sidak’s multiple comparison test. In line with previous studies, mice with test time frozen below 15% were removed from analysis [29, 72]. No mice were removed from the 0.5 mA acute nicotine fear conditioning study. In the 0.35 mA acute nicotine fear conditioning study we removed 4 mice from analysis, 3 male saline mice and 1 female saline mouse, due to test percent time frozen below 15% [29, 72]. We used a 15% time freezing criteria for contextual fear conditioning as initial percent time frozen below 15% we would be unable to determine extinction learning. The 15% minimum was chosen because when mice that undergo contextual fear conditioning under a similar shock intensity (0.57 mA), mice froze ∼ 15% of the time in a novel environment [73]. All data are expressed as mean ± SEM.

In experiment 2, chronic nicotine and spontaneous withdrawal fear extinction, statistical analyses were performed with GraphPad Prism 10.0 software package (GraphPad Software, CA) except for where otherwise stated. One male saline mouse and one male withdrawal mouse were removed for not meeting the 15% freezing threshold. The fear conditioning results were analyzed using three-way repeated measures ANOVA (*N* = 9–14 per sex*drug group), and time, drug, and sex were treated as factors. We observed main effects of time (F(1,65) = 964.3, *P* < 0.0001), sex (F(1,65)= 15.13, *P* < 0.001), and a time*sex interaction ( F(1,65)= 20.19, *P* < 0.0001) in contextual fear conditioning, with conditioning causing a robust increase in percent time frozen in all groups during the test, but males freezing more than their female counterparts in the test. Therefore, we separated the behavioral analysis based on sex to prevent baseline differences in conditioning from obfuscating potential differences in extinction learning. The fear conditioning results were then separated by sex and analyzed using a two-way repeated measures ANOVA, with time and drug treated as between subject factors.

The fear extinction results were analyzed via Graphpad using nonlinear segmental linear regression. Prior research in rodents indicates that 48 h is the peak withdrawal symptomology timepoint which correlates with diverse molecular endpoints, including transcriptomic changes [29, 67, 74, 75, 76]. Therefore, we evaluated if 48 h of withdrawal acted as inflection point in a segmental linear regression to differentially model extinction learning in the saline, nicotine, and withdrawal female and male mice separately. To determine whether the 48 h timepoint was a critical inflection point for our studies as well, we set the inflection point at extinction session 2 (E2) or 48 h of withdrawal as the constraint, and tested whether one line fit the saline, nicotine, and withdrawal groups, treating each replicate Y value as an individual point. The 48 h withdrawal timepoint represents a convergence of the peak behavioral withdrawal alterations and extensive genomic remodeling within neurocircuitry directly related to the behavior [29, 67, 74, 75]. If one segmental linear regression model did not fit all three groups, we tested each treatment group individually to assess if the slope from PT to E2 and from E2 to E5 were statistically different from 0, to examine if extinction learning was occurring during each time frame to assess change over time within treatment groups.

For all biochemical analysis outliers were determined using the Grubb’s outlier test with *P* = 0.05. The nAChR binding data was analyzed using a one-way ANOVA with drug treatment as the between subject factors. Results from RT-PCR were analyzed by a two-way ANOVA with drug and sex as factors, followed by Sidak’s multiple comparison tests if a sex*drug interaction was observed. If only a main effect of drug was observed a column comparison was conducted exploring differences in expression between saline, nicotine, and withdrawal groups without consideration of sex as a variable and using Sidak’s multiple comparison test.

Following this analysis, we next assessed individual transcriptomic changes in the genes of interest for their predictive value of behavioral outcomes in the delay of extinction learning, thereby supporting the biological importance of the observed behavioral differences. We used GraphPad Prism to generate nonlinear segmental linear regression setting the inflection point at E2 as the constraint for each individual mouse. The slopes from PT to E2 (slope 1) and E2 to E5 (slope 2) were used to calculate the change in slope through extinction (slope 2 - slope 1) as a model for delay in extinction learning. In order to assess changes in the genes of interest for their predictive value of delays of extinction learning, we performed a stepwise linear regression including gene expression as our independent variables and change in extinction slope as our dependent variable. We used SPSS Statistics software package 29 (IBM, NY) to perform linear regressions treating gene expression data as the independent variables and change in slope as the dependent variable. We used the stepwise method and F value of 2 for entry and 1 for removal, to generate models to predict delays in fear extinction (change in slope) based on dorsal or ventral gene expression. Sexes were analyzed separately for model generation. Models were generated including either all Dhip or Vhip gene expression data. The observed change in slope values were plotted against predicted and tested in Prism using linear regression for slopes that were statistically different from 0. All data in bar graphs are expressed as mean ± SEM, all data in line graphs are expressed as mean with a 95% CI.

## Results

### Sex differences in sensitivity to fear conditioning are modulated by Acute Nicotine

**Fig. 1:**
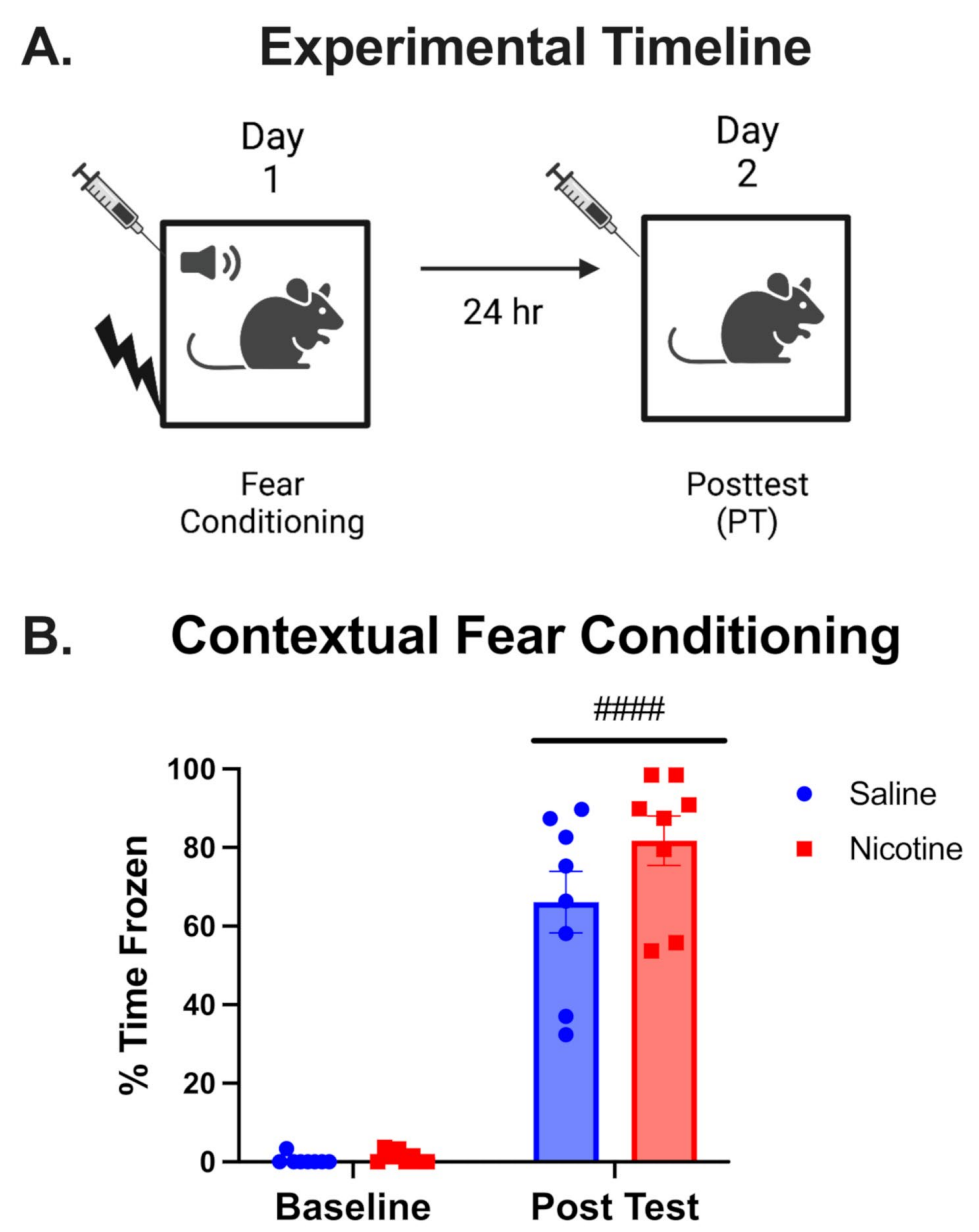
Impacts of acute nicotine on contextual fear conditioning. (**A**) Experimental timeline for acute nicotine’s impacts on contextual fear conditioning. Syringes represent i.p. injection of nicotine or vehicle control (saline) 10 min prior to conditioning or test. (**B**) Bar graph shows impact of acute nicotine (0.5 mg/kg i.p.) on contextual fear conditioning with a 0.5 mA shock intensity (US) measured in percent time frozen. Both males and females were used [*n* = 8 per treatment group] but due lack of nicotine effect was not disambiguated on the basis of sex [*n* = 4 per sex treatment group; error bars are SEM; #### - *P* < 0.0001 main effect of time]

Contextual Fear Conditioning is a well-established hippocampus-dependent learning and memory task [37, 38, 77]. A plethora of literature has demonstrated that acute nicotine immediately preceding conditioning increases percent time frozen in the test in contextual, but not cue, fear conditioning, in male subjects [27, 28, 40]. Therefore, we began testing the impacts of acute nicotine on contextual learning in male and female mice to validate our model prior to focusing on chronic nicotine and nicotine withdrawal’s impact on fear extinction. Basing our design on previous studies [39, 40], we administered acute nicotine (0.5 mg/kg) prior to both the conditioning and test days using a US of 0.5 mA in both male and female mice (Fig. 1.A). The 0.5 mA US caused a robust increase in time frozen in the test, with a main effect of time ( F(1,14) = 219.7, *P* < 0.0001). However, a ceiling effect precluded observation of any nicotine effects (Fig. 1.B). We next decreased our US to 0.35 mA and treated sex, time, and drug as a variables in a repeated measures 3-way ANOVA. We found main effects of time ( F(1,20) = 399.5, *P* < 0.0001) with conditioning causing an increase in percent time inactive in the test, and drug ( F(1,20) = 7.52, *P* = 0.0125), and interactions of time*sex (F(1,20) = 16.42, *P* = 0.0006) and time*drug ( F(1,20) = 4.572, *P* = 0.045). Post Hoc tests revealed that acute nicotine increased percent time frozen in males, but not females at this stimulus intensity, and saline treated males froze more than their female counterparts (Fig. S.1).

### Impacts of chronic nicotine and spontaneous withdrawal on contextual fear extinction

**Fig. 2:**
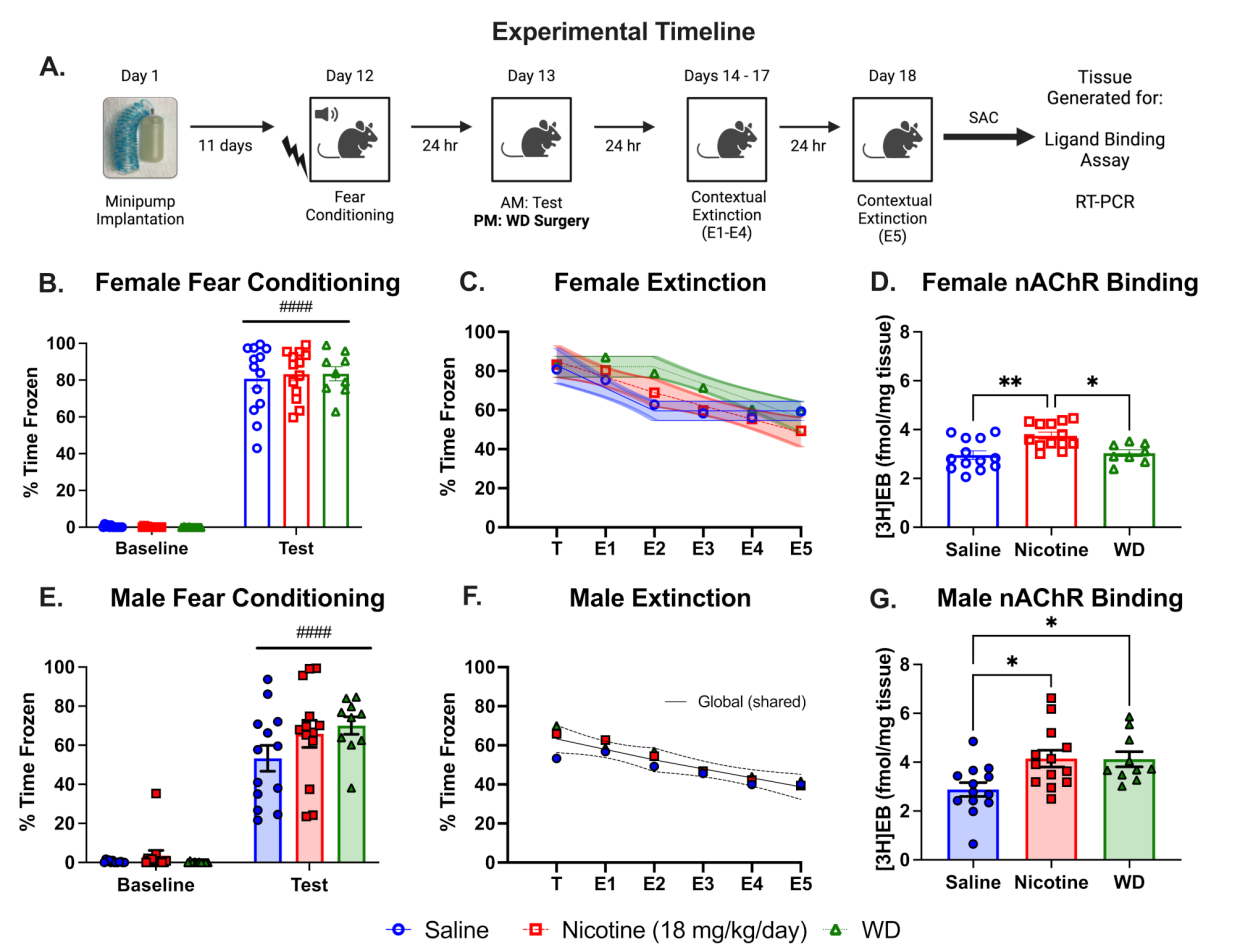
Chronic nicotine exposure and withdrawal contextual fear extinction. (**A**) Experimental timeline for chronic nicotine and nicotine withdrawal’s impacts on contextual fear extinction. (**B-D**) Graphs showing females data. (**B**) Bar graphs show impact of and chronic nicotine (18 mg/kg/day) on contextual fear conditioning in female mice with a 0.5 mA shock intensity (US) measured in percent time frozen. WD groups have not undergone surgery yet but highlights no differences in conditioning between mice marked for WD and nicotine mice. (**C**) Show contextual fear extinction graphs for saline, nicotine, WD females respectively measured in percent time frozen with 95% CI. Modeled lines are the segmental linear regression testing if slope 1 or slope 2 are statistically different than 0. (**D**) Cortex tissues from female mice treated with saline or nicotine in the contextual fear extinction experiments were used for homogenate-binding experiments with a saturating concentration of [^3^H]epibatidine ([^3^H]EB, 1.5 nM). (**E-G**) Graphs showing male data. (**E**) Bar graphs show impact of and chronic nicotine (18 mg/kg/day) on contextual fear conditioning in male mice with a 0.5 mA shock intensity (US) measured in percent time frozen. WD groups have not undergone surgery yet but highlights no differences in conditioning between mice marked for WD and nicotine mice. (**F**) Show contextual fear extinction graphs for saline, nicotine, WD males respectively measured in percent time frozen with 95% CI. Modeled line is testing if one segmental linear regression fit all three groups. (**G**) Cortex tissues from male mice treated with saline or nicotine in the contextual fear extinction experiments were used for homogenate-binding experiments with a saturating concentration of [^3^H]epibatidine ([^3^H]EB, 1.5 nM). [*n* = 9 to 13 per treatment; error bars are SEM; * - *P* < 0.05]

We next investigated the impacts of chronic nicotine and spontaneous withdrawal on extinction learning (Fig. 2.A). A two-way repeated measures ANOVA on female conditioning demonstrated a main effect of time ( F(1,32) = 1045, *P* < 0.0001). We observe no impact of chronic nicotine on fear conditioning in females (Fig. 2.B). The lack of nicotine effect was expected as numerous studies have found that the enhancing effects of acute nicotine on contextual fear conditioning disappear when treatment becomes chronic [27, 28, 74]. The lack of effect of chronic nicotine also highlights that any observed differences are not due to differences in conditioning. Radioligand binding to nAChRs confirmed receptor upregulation in the nicotine treated group ( F(2,31) = 8.188, *P* = 0.0014), a canonical sign of chronic nicotine exposure observed in both rodents and humans [78–80]. One withdrawal female mouse was removed from analysis as it was a significant outlier, which had nAChR levels of 6.81 fmol/mg of tissue. However, nAChR density had returned to baseline levels in the withdrawal group (Fig. 2.D). A segmental linear regression with an inflection point at E2 (48 h of WD) was applied to the saline, nicotine, and withdrawal (WD) female mice, which revealed that one curve did not adequately fit all three groups (F(6,201) = 2.412, *P* = 0.0284). The saline group had a slope from PT to E2 that was statistically different than 0 ( F(1,75) = 9.597, *P* = 0.0037), but the slope from E2 to E5 was not different from 0 ( F(1,75) = 0.6614, *P* = 0.4187)(Fig. 2.C). Both slopes in the chronic nicotine females from PT to E2 and E2 to E5 were statistically different from 0 (PT - E2:  F(1,75) = 7.412, *P* = 0.0081; E2 - E5:  F(1,75) = 12.18, *P* = 0.0008)(Fig. 2.C). The withdrawal group demonstrated a delayed extinction compared to their saline and nicotine counter parts (Fig. 2.C), where the slope from PT to E2, the first 48 h of withdrawal, was not statistically different from 0 ( F(1,51) = 1.002, *P* = 0.3216), but beyond the 48 h withdrawal timepoint, E2 to E5, the slope was statistically different from 0 ( F(1,51) = 12.90, *P* = 0.0007).

Similar to above, we observed a main effect of time in the male fear conditioning cohort ( F(1,33) = 262.2, *P* < 0.0001), but no statistically significant impact of drug (Fig. 2.E). There was a main effect of drug in the nAChR binding assay ( F(2,33) = 5.483, *P* = 0.0088), with increased [^3^H]epibatidine binding in cortical samples from nicotine treated male mice compared to their saline controls (Fig. 2.G). In contrast to female subjects, though, this nAChR upregulation persisted despite 5 days of nicotine withdrawal in male mice. However, when we applied a segmental linear regression with an inflection point at E2 the male saline, nicotine, and withdrawal mice, we found that one curve did include variance accounted for in all three groups (F(6,207) = 0.6287, *P* = 0.7072) (Fig. 2.F). Dorsal and ventral hippocampus samples from these animals were used for RT-PCR analysis outlined in the sections below.

We used a two-way ANOVA to investigate impacts of drug and sex on the change in slope value, and found an interaction of sex*drug treatment on the change in slope value ( F(2,65) = 3.187; *P* = 0.0478), with female saline mice having greater change in slope compared to withdrawal females and saline males, which further highlighted the sex differences in contextual learning (Fig. S.2). A change in slope greater than 0 was considered normal extinction in females as the saline controls had a decreased in percent time frozen followed by a plateau, while a change in slope less than 0 indicated a delay in extinction learning in females.

### Chronic nicotine and spontaneous withdrawal causes suppression of dorsal hippocampal gene expression

**Fig. 3:**
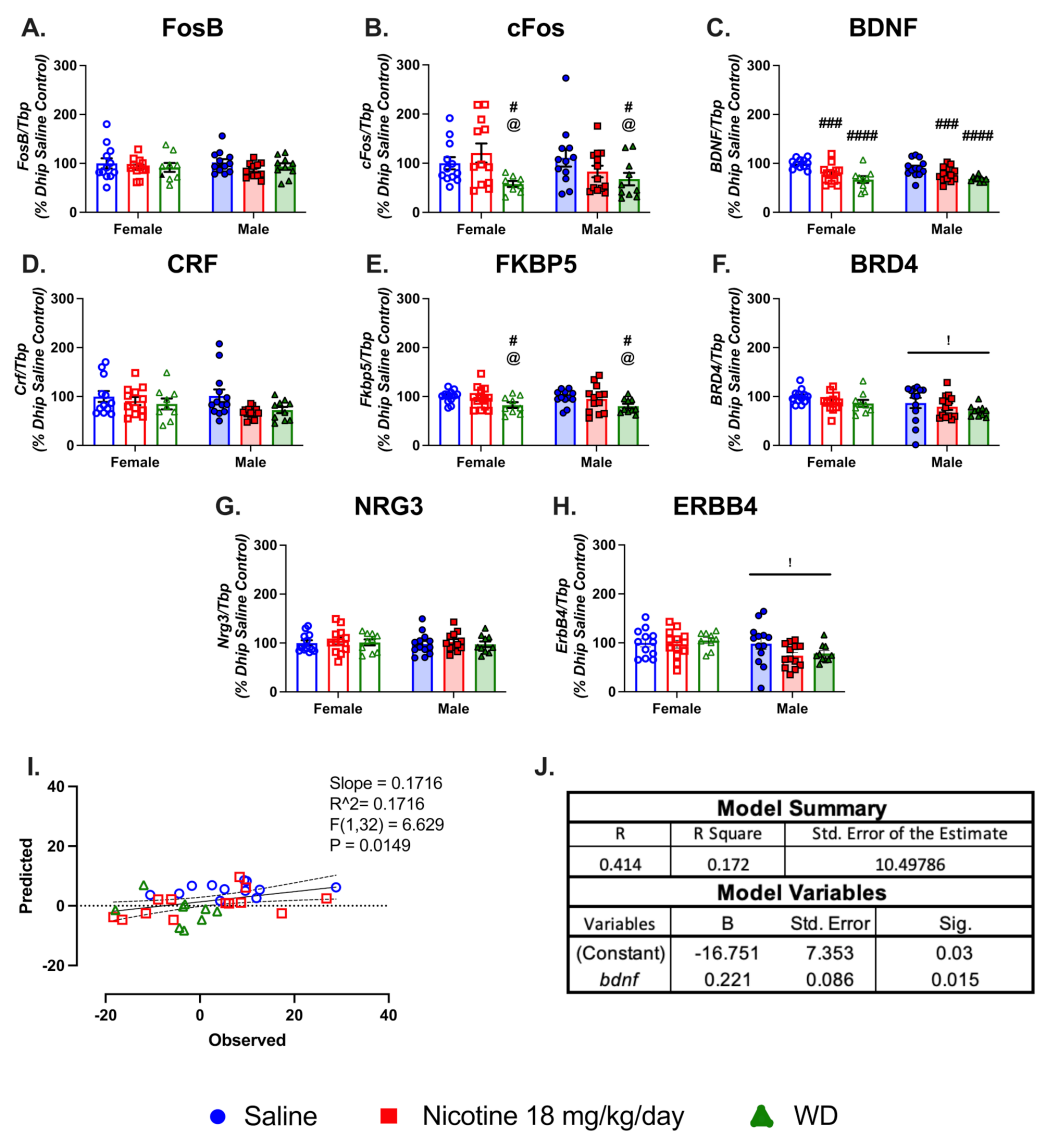
Impacts of sex on gene expression in the ventral hippocampus. (**A-H**) Bar graphs show qPCR analysis of gene proxies for neuronal function and plasticity, PTSD related genes, and neuregulin signaling pathway genes mRNA expression in the dorsal hippocampus of saline, chronic nicotine, and withdrawal treated female and male mice. (**I**) The graphed predicted versus observed values in change of slope for the female mice. Predicted values were generated using female dorsal hippocampal gene expression values and observed change in slope values in SPSS statistics. Statistics presented on graph are the linear regression between predicted and observed change in slope values generated with GraphPad Prism with 95% CI. (**J**) The table are the coefficients used in the female gene expression model to predict changes in slope. *bdnf*, Brain Derived Neurotrophic Factor [*n* = 9 to 14 per region treatment; error bars are SEM; column comparison of drug compared to saline: # - *P* < 0.05, ### - *P* < 0.001, #### - *P* < 0.0001; column comparison of drug compared to nicotine: @ - *P* < 0.05; main effect of sex: !<0.05]

We next evaluated dorsal and ventral hippocampal samples for transcriptional changes in the expression of *fosb*, *cfos*, and *bdnf* as a proxy for neuronal activation and plasticity changes in the Dhip. Our qPCR analysis found that drug and sex had no significant impacts on *fosb* expression in the Dhip (Fig. 3.A). However, *cfos* mRNA expression displayed a main effect of drug with WD mice having lower expression compared to saline and chronic nicotine mice ( F(2,63) = 4.147, *P* = 0.0203; Fig. 3.B). Similarly, we observe a main effect of drug ( F(2,64) = 16.36, *P* < 0.0001), but not sex on *bdnf* mRNA expression in the Dhip, with both chronic nicotine and withdrawal mice having lower *bdnf* expression in the Dhip compared to saline controls (Fig. 3.C). We next examined nicotine treatment’s impact on the expression of *crf*, *fkbp5*, and *brd4* which are genes that have been implicated in both preclinical models of PTSD and clinical populations diagnosed with PTSD, a condition characterized by exaggerated fear memory consolidation [53, 56, 57]. Nicotine treatment significantly impacted expression of both *crf* and *fkbp5.* Main effects of drug were observed in both cases (*crf*: F(2,63)  = 3.726, *P* = 0.0296; *fkbp5*:  F(2,65) = 4.914, *P* = 0.0103). Column comparisons for *crf* found no statistically significant differences between treatment groups (Fig. 3.D), but for *fkbp5* spontaneous withdrawal caused a down regulation in gene expression relative to both saline and chronic nicotine. (Fig. 3.E). We also found a main effect of sex ( F(1,64) = 5.085, *P* = 0.0276) on *brd4* mRNA expression in the Dhip, with males having lower expression compared to females (Fig. 3.F). We next evaluated *nrg3* and *erbb4* mRNA levels, which specifically have been linked to hippocampal dysfunction during smoking cessation in animals and humans [58, 59, 78, 71, 81]. However, there were no main drug effects on *nrg3* or *erbb4* expression at this time point (Fig. 3.G, H). There was a main effect of sex ( F(1,64) = 6.273, *P* = 0.0148) on *erbb4* expression, with males having lower expression compared to females (Fig. 3.H).

We then used SPSS statistics to perform linear regressions of all female Dhip gene expression with female change in slope values, and found a statistically significant model to predict delays in extinction learning in females (R^2^ = 0.1716;  F(1,32) = 6.629, *P* = 0.015), with Dhip *bdnf* expression being the only gene with statistically significant predictive validity (Fig. 3.I-J). Performing the same analysis with male gene expression and change in slope values, we found a statistically significant model to predict delays in extinction learning in males (R^2^ = 0.136;  F(1,34) = 5.33, *P* = 0.027). Dhip *cfos* expression was the only gene with statistically significant predictive validity and this model was not predictive of female change in slope (Fig S.5 A-C).

### Nicotine and withdrawal cause sex specific changes in ventral hippocampal gene expression

**Fig. 4:**
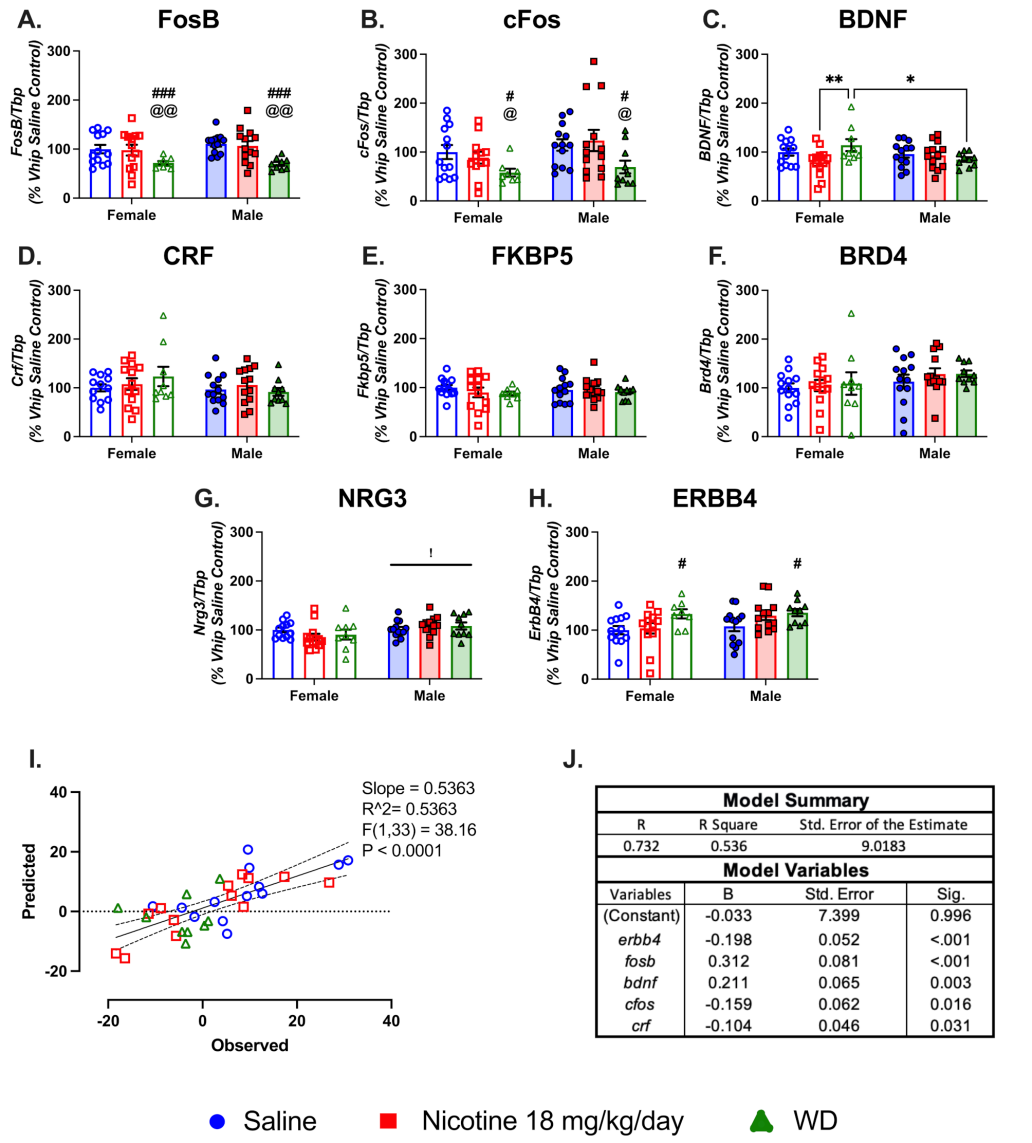
Impacts of sex on gene expression in the ventral hippocampus. (**A-H**) Bar graphs show qPCR analysis of gene proxies for neuronal function and plasticity, PTSD related genes, and neuregulin signaling pathway genes mRNA expression in the ventral hippocampus of saline, chronic nicotine, and withdrawal treated female and male mice. (**I**) The graphed predicted versus observed values in change of slope for the female mice. Predicted values were generated using female ventral hippocampal gene expression values and observed change in slope values in SPSS statistics. Statistics presented on graph are the linear regression between predicted and observed change in slope values generated with GraphPad Prism with 95% CI. (**J**) The table are the coefficients used in our generated model to predict changes in slope. *erbb4*, Erb-B2 Receptor Tyrosine Kinase 4; *fosb*, FosB Proto-Oncogene, AP-1 Transcription Factor Subunit; *bdnf*, Brain Derived Neurotrophic Factor; *cfos;* Fos Proto-Oncogene, AP-1 Transcription Factor Subunit; *crf*, Corticotropin Releasing Hormone; [*n* = 9 to 14 per region treatment; error bars are SEM; drug sex interaction: * - *P* < 0.05, ** - *P* < 0.01; column comparison of drug compared to saline: # - *P* < 0.05, ## - *P* < 0.01; column comparison of drug compared to nicotine: @ - *P* < 0.05, @@ - *P* < 0.01; main effect of sex: !<0.05]

We next explored how chronic nicotine treatment and spontaneous withdrawal impacted gene expression in the Vhip. Similar to the Dhip, we observed the largest impacts of drug on our gene proxies for neuronal activation and plasticity changes with limited impact on PTSD and smoking cessation outcome linked genes. This was most evident in *fosb* mRNA levels, where we found main effect of drug ( F(2,65) = 8.816, *P* = 0.0004), with column comparisons showing withdrawal reduced expression in both sexes compared to saline and chronic nicotine (Fig. 4.A). The impacts of nicotine treatment on *cfos* expression mirrored *fosb*, with nicotine and withdrawal reducing expression of *cfos* in both sexes compared to saline and chronic nicotine (main effect drug:  F(2,65) = 4.924, *P* = 0.0102; Fig. 4.B). Vhip expression of *bdnf* increased in the females only during withdrawal compared to nicotine treatment in females and their male withdrawal counterparts (drug*sex interaction:  F(2,65) = 3.494, *P* = 0.0362) (Fig. 4.C). While we saw drug treatment heavily influence expression of proxies for neuronal activation in Vhip, we found no impact of drug or sex on PTSD-linked mRNA (Fig. 4.D-F). In the case of the smoking cessation outcome linked genes, there was a main effect of sex on *nrg3* ( F(1,64) = 7.121, *P* = 0.0096) with males having higher expression than females (Fig. 4.G). Similar to previous work from our group, we found a main effect of drug treatment on *erbb4* expression (F(2, 64 ) = 4.769, *P* = 0.0177), with nicotine withdrawal causing an increase in mRNA levels in the Vhip compared to saline controls (Fig. 4.H) [58, 81]. To directly compare alterations in the Vhip transcriptome with behavior, we next evaluated correlations between gene expression and behavioral endpoints.

We performed a linear regression of all female Vhip gene expression with female change in slope values, and found a statistically significant model to predict delays in extinction learning in females (R^2^ = 0.5363;  F(5,29) = 6.709, *P* < 0.001), with Vhip expression of *erbb4*, *fosb*, *bdnf*, *cfos*, and *crf* having statistically significant predictive validity (Fig. 4.I-J). The predicted change in slope values significantly correlated to the observed values in females. While we did not observe behavioral differences in treatment groups of the males, we performed the same linear regression using the male change in slope and all male Vhip gene expression data to validate that female gene expression was not predictive in males with a different variable coefficient. We found a statistically significant model to predict delays in extinction learning in males (R^2^ = 0.3037;  F(3,32) = 4.651, *P* = 0.008), with expression of Vhip *fkbp5*, *crf*, and *bdnf*, and this model was not predictive of female change in slope (Fig. S.5D-F).

## Discussion

This study assessed sex differences in response to acute and chronic nicotine treatment in contextual fear conditioning and evaluated the transcriptomic alterations associated with these effects. Our findings also highlighted sex specific effects of nicotine withdrawal during fear extinction. We were able to replicate acute nicotine induced increased percent time frozen in the test in male mice using a US of 0.35 mA [39–41]. We observed an increase in the nicotine treated females, but it was not statistically significant, which is likely due to saline control females having a higher percent time frozen in the test than saline males, a trend we continued to observe in both contextual fear conditioning assays. Previous studies have found that chronic nicotine exposure and spontaneous withdrawal caused deficits in contextual fear extinction in male mice [29, 49]. However, we report that that chronic nicotine exposure and spontaneous withdrawal had no impact on male contextual fear extinction. Further, nicotine withdrawal caused delays in female extinction learning, a finding that to our knowledge has not been previously reported. Interestingly, female delays in extinction learning were predicted by specific transcriptional gene expression in a subregion-specific manner, with neuronal activity and smoking cessation genes in the Vhip strongly predicting extinction learning. This information could be utilized in the future for individualized treatment of smoking cessation in women, especially those with co-morbid PTSD. Prolonged exposure therapy is an effective psychotherapy for treatment of PTSD recommended by multiple professional organizations [82, 83]. Prolonged exposure therapy tries to promote emotional processing through exposure to trauma-related stimulus or cues, via either real life exposure to falsely assumed dangerous contexts or exposure to images or memories related to the traumatic event [84]. Contextual fear extinction is similar, as mice learn that the previously aversive environment is now safe. Understanding how chronic nicotine and withdrawal impact this safety learning and identifying potential molecular underpinnings of the mechanism of this safety learning can identify novel targets for women with comorbid PTSD and nicotine use disorder to address cognitive deficits experienced during nicotine withdrawal and improve outcomes for exposure therapy treatment for PTSD.

### Acute Nicotine Impact on Contextual Fear Conditioning

The impacts of acute nicotine on hippocampally mediated learning and memory tasks are well established. When examining the acute effects of nicotine in nonsmoking individuals, a meta-analysis showed that acute nicotine improved response time in the cognitive domains of attention and episodic memory [85]. Specifically in rodents, multiple doses of systemic acute nicotine (0.045 mg/kg, 0.09 mg/kg, 0.125 mg/kg, 0.25 mg/kg, 0.5 mg/kg) prior to contextual fear conditioning have been reported to increase time frozen in the test under multiple different shock intensities (0.17 mA, 0.35 mA, 0.50 mA, 0.57 mA) [27, 28, 39, 40, 86]. Previous studies have also shown that nicotine injections are necessary prior to both conditioning and contextual testing for enhancement of contextual fear conditioning [39, 40]. These studies found that nicotine treatment only prior to the conditioning session or only prior to the contextual testing did not impact percent time inactive in male or female mice, suggesting that acute nicotine treatment is not solely impacting the formation or recall of the fear memory [39, 40]. Rather it is acute nicotine prior to both sessions that synergistically impacts the formation, consolidation, and retrieval of the fear memory.

While the impact of acute nicotine on contextual fear conditioning is well established [27, 28, 39, 40, 86], many of these studies contained only male mice. One previous study from Gould (2003) found that an acute nicotine dose of 0.5 mg/kg increased time frozen in the test when administered prior to training and test in both male and female mice, with no effects of sex observed [39]. In the present study, the nicotine dose of 0.5 mg/kg did not elicit enhancement of contextual fear conditioning in male and female mice when using a 0.5 mA shock US, perhaps due to strain differences regarding stimulus sensitivity. However, using a lower intensity US (0.35 mA), we found that acute nicotine increased fear memory formation and recall in males, as previously reported [39, 40], but not in female mice.

While we observed an increase in percent time freezing in the test of acute nicotine treated female mice, the increase failed to reach statistical significance. We believe this was due to a task-related ceiling effect, as female mice had higher freezing than males treated with acute saline, and it is possible that increasing *N* or decreasing the shock intensity would result in statistical significance. However, this may also be a sexually dimorphic response to acute nicotine in contextual fear memory. While previous human studies have found acute nicotine enhanced aspects of hippocampally dependent memory, sex differences were not investigated [85, 87, 88]. There are numerous sex differences in hippocampal morphology and function, with large impacts of the estrus cycle in females (for review see [89, 90]). While previous rodent studies have found no impact of estrus cycle [91, 92] or ovariectomy [93] on freezing in the test, we could have observed an interaction of nicotine and estrus cycle phase, given the interplay between hormones and acetylcholine signaling previously observed in female rats [94]. Another possibility relates to differences in experimental design, such as pre-exposure to conditioning context, shock intensity, and length of test [92, 95, 96]. For example, Clark et al. found that males had greater contextual fear conditioning compared to females only at the high shock intensity of 0.8 mA, while no sex differences were observed at the lower 0.6 mA [96]. Interestingly, Keiser et al. had almost identical experimental conditions as the Clark et al. 0.8 mA shock intensity experiment but found that females had greater fear conditioning [92]. A notable difference between these two experiments is that Clark et al. included a tone conditioned stimulus, that was absent in the Keiser et al. experiment [92, 96]. Both Clark et al. and Matsuda et al. found that when mice are pre-exposed to the conditioning context there are no observed sex-differences in contextual fear conditioning [92, 95], suggesting that pre-exposure to the context heavily influences experimental outcomes. Mouse studies are often in different inbred strains of mice, indicating a potential role for genetic diversity in these observations. The referenced mouse studies use C57b6 mice, while our studies utilized an F1 hybrid of two inbred strains, C57b6 and 129 A/J. A previous study investigating 8 mouse strains found strain dependent effects of acute nicotine on contextual fear conditioning, with both C57b6 and 129/SvEe having increases, but C57b6 were more sensitive, i.e. required lower dose of nicotine to elicit increase in freezing, and had lower drug naïve freezing compared to 129/SvEe, but this was conducted in only males [97]. We may have observed a strain by sex interaction, with females in our hybrid F1 strain being less sensitive to acute nicotine’s enhancing effects on contextual fear conditioning compared to their male counter parts or females of other strains.

### Sex and chronic nicotine impacts on contextual fear extinction

Male and female smokers present with deficits in both physiological and subjective measures of contextual fear inhibition [25] and impaired ability to discriminate danger and safety cues in differential fear conditioning [26]. Additionally, previous studies in mouse models found that chronic nicotine exposure during contextual fear conditioning and extinction resulted in fear extinction deficits in males but not females [49]. While we were able to validate previous findings that chronic nicotine had no impact on contextual fear conditioning [27, 28], we were not able to replicate previous findings reporting chronic nicotine and nicotine withdrawal induced deficits in extinction behavior in male mice [29, 49], despite successfully eliciting extinction of fear memory in both sexes. One possible explanation is genetic influences, as B6129SF1/Tac male mice did not show nicotine withdrawal induced deficits in contextual fear conditioning, while C57BL/6NTac male mice had robust impairment [98], and previous extinction studies were conducted in C57BL/6NTac [29, 49]. An alternative explanation may relate to differences in nicotine delivery, given our intermittent delivery system compared to their continuous delivery of nicotine. In contrast, we found robust delays in extinction learning in female mice withdrawn from nicotine. Interestingly, this does not map to clinical data, where men tend have greater cognitive-related impairments during nicotine withdrawal [21, 22]. Nicotine withdrawal may specifically impact females in extinction of an aversive memory associated with fear, as previous studies have shown chronic nicotine improved performance of female rats in the radial arm maze [99], a spatial learning and memory task in service of receiving a rewarding stimulus of food [100]. Clinical data supports this supposition, as women (1) are more likely to develop PTSD [101], (2) report larger increases in anxiety score during nicotine withdrawal [20], and (3) the relationship between PTSD and smoking relapse due to withdrawal is stronger in females [12, 102].

While we did not track estrus cycle in our female mice, it is possible that circulating sex hormones could have influenced conditioning and/or extinction learning. The increased conditioning response observed in females is somewhat surprising as it is typically assumed that male rodents have stronger memory consolidation and recall in fear conditioning than females [103]. Current literature on the impact of sex on contextual fear conditioning is mixed. Rat studies have more consistently found that females have weaker fear memory consolidation in a 24 h to 48 h delayed test [91, 104, 105]. Others have found that male and female, ovariectomized or sham control, rats froze the same amount in a 24 h delayed test [93]. Prior studies in mice have reported both a significant effect of sex, with females freezing more than males and no differences in female conditioning due to estrous cycle [92], and females freezing less than males [96], as well as no impact of sex on contextual fear conditioning [95]. Contextual fear extinction using rats found that females have improved fear extinction compared to males, but this was eliminated after ovariectomy, with no baseline differences in conditioning. This was rescued when ovariectomized rats were administered estrogen [93], highlighting the potential role of the estrus cycle in mediating contextual fear extinction. Furthermore, female rats that underwent contextual fear conditioning during the estrus and proestrus phases had increased contextual fear extinction compared to male rats and female rats in the diestrus phase [91]. However, when exploring contextual fear extinction in mice Matsuda et al. found sex differences in percent time freezing within extinction session. Females showed more resistance to extinction learning and ovariectomy caused reduction in extinction learning, but these differences were observed within specific extinction sessions and were not found across extinction sessions [95]. Taken together estrus cycle phase during conditioning could have directly impacted or interacted with nicotine treatment to influence conditioning and or extinction and should be further investigated in future studies.

Regarding chronic nicotine’s impact on fear memory consolidation and retrieval, we did not observe any differences in the contextual fear conditioning between chronic nicotine groups and saline controls, a finding previously observed [27, 28], suggesting chronic nicotine did not impact short term retrieval of fear memory. While it is possible that chronic nicotine treatment did impact long term retrieval, this seems unlikely. Tumolo et al. found that mice treated with chronic nicotine after fear conditioning did not have altered contextual fear recall when tested 13 days after conditioning [106]. However, chronic nicotine treatment had sex specific effects on spontaneous recovery of contextual fear when administered after contextual fear extinction [106], with chronic nicotine decreasing recovery in male and increasing recovery in females. Further studies are needed to elucidate how chronic nicotine during conditioning impacts extinction learning once chronic treatment has stopped and post withdrawal behavioral phenotypes and how chronic nicotine and withdrawal specifically impacts extinction learning after drug naïve conditioning.

### Sex and Subregional Specific impacts of Nicotine and Withdrawal during Contextual fear extinction on Gene expression in the Hippocampus

Transcriptional activation can serve as a marker of circuit activity as well as an indication of drug effects on neuroplasticity. In this study, we utilized quantitative RT-PCR to detect alterations in transcriptional expression of specific indicator genes to probe both of these measures following nicotine withdrawal in male and female subjects. We linked gene expression data to our behavioral model via our change in slope calculation. This approach more accurately modeled the behavior observed in the female extinction learning than a traditional slope or percent change, because while all treatment groups had similar percent time frozen in the test and E5, we observed treatment specific extinction learning patterns in our paradigm.

First, we investigated sex and drug effects on dorsal and ventral hippocampal circuit activity using two canonical immediate early genes, FosB Proto-Oncogene (*fosb*) and Fos Proto-Oncogene (*cfos*), in addition to a predominant downstream effector of immediate early genes activity following neuronal activity, namely Brain Derived Neurotrophic Factor (*bdnf*) [50–52]. In the dorsal hippocampus, which subserves the contextual elements of episodic memory, we observed robust effects of nicotine treatment on both *cfos* and *bdnf* RNA expression in both sexes. However, Dhip *bdnf* expression was the only statistically significant predictor of delays in extinction learning in females, suggesting that RNA expression in the Dhip of the 8 genes we investigated was not accounting for the variance observe in female extinction. In the ventral hippocampus, which is more closely aligned with the affective coding of contextual memory [43], we report mice undergoing nicotine withdrawal had a significant repression of both *fosb* and *cfos*. However, unlike our findings in the Dhip, we observed a treatment*sex effect on *bdnf*, where female Vhip samples showed increased *bdnf* expression during withdrawal as compared to nicotine treatment. Prior work from our lab has shown that CREB activity in the ventral hippocampus both (1) results in *bdnf* transcription and (2) impairs behavioral responding in the contextual fear conditioning paradigm [71]. This is congruent with our current observations indicating an increase in *bdnf* expression co-occurs with deficits in the extinction of fear memory. Unlike in the Dhip, all three genes linked to neuronal activation and plasticity were statistically significant predictors of delays in extinction learning. Based on this model, lower expression of *fosb* and *bdnf* expression was predictive of larger delays in extinction learning, as both variable coefficients were positive and a change in slope greater than 0 was considered normal extinction in females. In contrast, we found the opposite in *bdnf* expression, with the withdrawal females having higher expression than their nicotine counterparts. Additionally, our model found that lower *cfos* expression in the Vhip would predict a shorter delay in extinction learning, as the *cfos* variable coefficient was negative. While our model was exploratory and did not map directly to all of our observed treatment effects in qPCR data, it highlighted that differences in Vhip neuronal activity of females were more predictive of dysfunction in contextual fear extinction learning than their male counterparts.

In addition to its use as a marker of cellular activity and neuroplasticity, profiling transcriptional activation can result in a better understanding of how psychiatric conditions with overlapping endophenotypes may also have overlapping mechanistic underpinnings. With this in mind, we queried the transcriptional status of three genes associated with post-traumatic stress disorder [53–57], which is characterized by impaired extinction of fear memory [107]. Corticotropin-releasing factor (CRF) has a well-established role in stress response and was increased in cerebral spinal fluid of individuals diagnosed with PTSD [53–55]. We found that nicotine treatment caused suppression of *crf* mRNA expression in the Dhip of both sexes, but only caused deficits in female extinction learning. Similarly, nicotine treatment caused a decrease in Dhip FK506-binding protein 5 (*fkbp5*) RNA, which is a co-chaperon of the glucocorticoid receptor complex that prevents the complex from translocating to nucleus [108]. A suppression of this protein could have lead to an increase in glucocorticoid signaling, possibly in response to nicotine treatment induced changes in *crf* we observed. Despite the impacts of drug treatment, neither *fkbp5* or *crf* expression in the Dhip was predictive of delays in extinction learning in females, but more tests are needed to delineate possible contributions of hippocampal *crf* and or *fkbp5* expression in nicotine withdrawal endophenotypes.

We also investigated two genes highly associated with hippocampal dysfunction due to nicotine withdrawal. Both *nrg3* and *erbb4* are involved in the Neuregulin Signaling Pathway, which has an important role in the development of the CNS and mediates the stabilization of synapses and synaptic plasticity in adulthood [109–111]. Neuregulin 3 (*nrg3*) exclusively binds to ErbB4-containing receptors (*erbb4*) [112, 113], and these genes have been associated with smoking cessation outcomes in two independent cohorts of smokers [58, 59]. We replicated and extended to a longer withdrawal period findings from previously published work whereby chronic nicotine and withdrawal caused an increase in *erbb4* mRNA expression in the Vhip [58]. We also observed sex specific expression differences in both of these genes, which correlates with sex-ratio differences in the conditions most highly associated with genetic alterations in these genes, such as schizophrenia and ADHD [114, 115]. Additionally, expression of *erbb4* within the ventral hippocampus of females in our treatment paradigm was predictive of change in slope of extinction learning, where higher ventral hippocampal *erbb4* expression was associated with female delays in fear memory extinction. This was an interesting corollary from previous work in our lab demonstrating that increased ventral hippocampal *erbb4* expression mechanistically underlies increased anxiety-like behavior during nicotine withdrawal [81].

## Conclusion

The present work suggests a potential sex specific impact of nicotine withdrawal during extinction learning, specifically during contextual fear extinction, with early evidence for discrete biological sex differences in sub-regions of the hippocampus resulting in sex specific delays in extinction learning. While drug treatment largely caused similar changes in gene expression in the Dhip and Vhip, we only observed delays in extinction learning in females. Expression of all genes used as proxies for neuronal function and *erbb4*, a smoking cessation outcome related gene, in the Vhip were predictors of delays in female extinction learning. This is a surprising finding given the Vhip is typically associated with affective responding, while the Dhip is traditionally considered to mediate contextual learning and memory [43]. While these predictive models were exploratory, our finds highlighted a potential sex differences in the hippocampal circuity, as nicotine treatment induced Vhip dysfunction appeared to be predicative of the female specific nicotine withdrawal induced deficits in extinction learning. A greater understanding of these dichotic effects may lead to more positive outcomes regarding smoking cessation when stratified by sex, especially in women who have comorbid psychiatric conditions characterized by exaggerated fear memory consolidation such as PTSD [53, 56, 57].

## Electronic supplementary material

Below is the link to the electronic supplementary material.

Supplemental Figure 1: Impacts of Acute Nicotine on Contextual Fear Conditioning at a Lower Shock Intensity. (**A**) Experimental timeline for acute nicotine’s impacts on contextual fear conditioning. Syringes represent i.p. injection of nicotine or vehicle control (saline) 10 minutes prior to conditioning or test. (**B**) Bar graph shows impact of sex and acute nicotine (0.5 mg/kg i.p.) on contextual fear conditioning with a 0.35 mA shock intensity (US) measured in percent time frozen. [*n* = 5 to 8 per sex treatment group; error bars are SEM; main effect of time: ####<0.0001, interaction: **P < 0.01, **** - *P* < 0.0001]

Supplemental Figure 2: Impacts of Nicotine Treatment on Change in Extinction Rate During Fear Extinction. Bar graph show impact of sex and nicotine treatment on contextual fear extinction measured in change in slope. [*n* = 9 to 14 per treatment; error bars are SEM; * - *P* < 0.05, ** - *P* < 0.001]

Supplemental Figure 3: Modeling Delays in Fear Extinction of Male Mice Using Female Dorsal Hippocampal Gene Expression. (**A**) The graphed predicted versus observed values in change of slope for the male mice using the model generated using female dorsal hippocampal gene expression data. Predicted values were generated using female dorsal hippocampal gene expression values and observed change in slope values in SPSS statistics. Statistics presented on graph are the linear regression between predicted and observed change in slope values generated with GraphPad Prism with 95% CI. While the predicted values significantly correlated to the observed values of change in slope if females, it was not predictive when using male Dhip gene expression values. (**B**) The table are the coefficients used in the female gene expression model to predict changes in slope. *bdnf*, Brain Derived Neurotrophic Factor [*n* = 10 to 13 per treatment]

Supplemental Figure 4: Modeling Delays in Fear Extinction of Male Mice Using Female Ventral Hippocampal Gene Expression. (**A**) The graphed predicted versus observed values in change of slope for the male mice using the model generated using female ventral hippocampal gene expression data. Predicted values were generated using female dorsal hippocampal gene expression values and observed change in slope values in SPSS statistics. Statistics presented on graph are the linear regression between predicted and observed change in slope values generated with GraphPad Prism with 95% CI. The model generated using female Vhip gene expression data was not predictive of change in slope in males. (**B**) The table are the coefficients used in our generated model to predict changes in slope. *erbb4*, Erb-B2 Receptor Tyrosine Kinase 4; *fosb*, FosB Proto-Oncogene, AP-1 Transcription Factor Subunit; *bdnf*, Brain Derived Neurotrophic Factor; *cfos;* Fos Proto-Oncogene, AP-1 Transcription Factor Subunit; *crf*, Corticotropin Releasing Hormone

Supplemental Figure 5: Modeling Delays in Fear Extinction Using Male Dorsal and Ventral Hippocampal Gene Expression. While we did not observe behavioral differences in treatment groups of the males, we performed the same linear regression using the male change in slope and all male Dhip (**A-C**) or all male Vhip (**D-F**) gene expression data to validate the same genes observed in females are not predictive in males with a different variable coefficient. (**A**) The graphed predicted versus observed values in change of slope for the male mice using the model generated using male dorsal hippocampal gene expression data. (**B**) The graphed predicted versus observed values in change of slope for the female mice using the model generated using male dorsal hippocampal gene expression data. Predicted values were generated using male dorsal hippocampal gene expression values and observed change in slope values in SPSS statistics. (**C**) The table are the coefficients used in our generated model to predict changes in slope using male dorsal hippocampal gene expression. *cfos;* Fos Proto-Oncogene, AP-1 Transcription Factor Subunit. (**D**) The graphed predicted versus observed values in change of slope for the male mice using the model generated from male ventral hippocampal gene expression data. (**E**) The graphed predicted versus observed values in change of slope for the female mice using the model generated from male ventral hippocampal gene expression data. Predicted values were generated using male ventral hippocampal gene expression values and observed change in slope values in SPSS statistics. (**F**) The table are the coefficients used in our generated model to predict changes in slope using male dorsal hippocampal gene expression. *Fkbp5*; *fkbp5*, FK506-Binding Protein 5; *crf*, Corticotropin Releasing Hormone; *bdnf*, Brain Derived Neurotrophic Factor; Transcription Factor Subunit. Statistics presented on graph are the linear regression between predicted and observed change in slope values generated with GraphPad Prism with 95% CI

## Data Availability

The raw data that support these findings are available upon request.
