# Supplementary figures and images for: Sex differences in contextual fear conditioning and extinction after acute and chronic nicotine treatment
Source: Biol Sex Differ. 2024 Oct 31;15:88. doi: 10.1186/s13293-024-00656-6 (PMC11529327; doi:10.1186/s13293-024-00656-6)

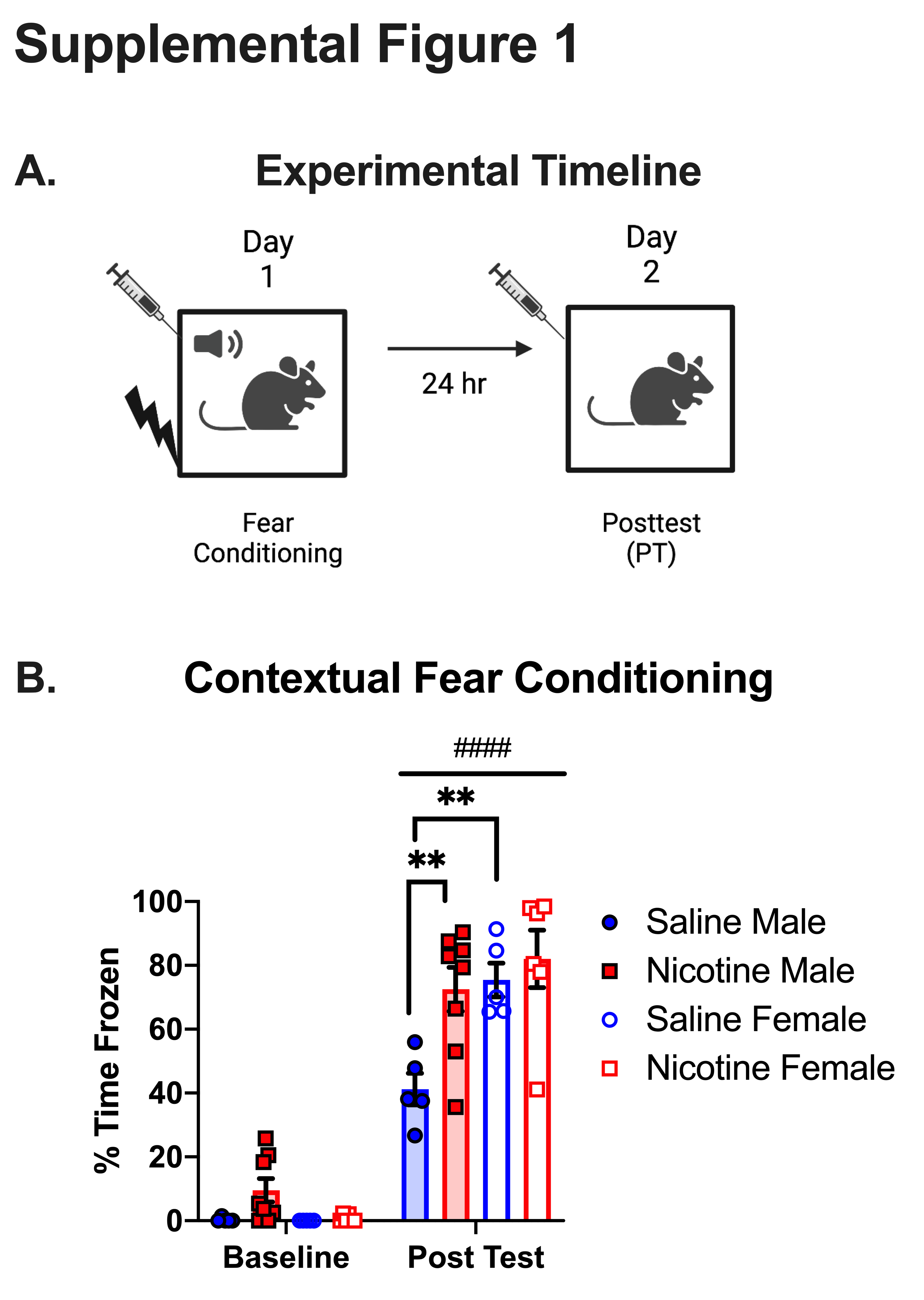

Supplement: Supplementary file 1 — Supplemental Figure 1: Impacts of Acute Nicotine on Contextual Fear Conditioning at a Lower Shock Intensity. (A) Experimental timeline for acute nicotine’s impacts on contextual fear conditioning. Syringes represent i.p. injection of nicotine or vehicle control (saline) 10 minutes prior to conditioning or test. (B) Bar graph shows impact of sex and acute nicotine (0.5 mg/kg i.p.) on contextual fear conditioning with a 0.35 mA shock intensity (US) measured in percent time frozen. [n = 5 to 8 per sex treatment group; error bars are SEM; main effect of time: ####<0.0001, interaction: **P < 0.01, **** - P < 0.0001] [file 13293_2024_656_MOESM1_ESM.tiff]

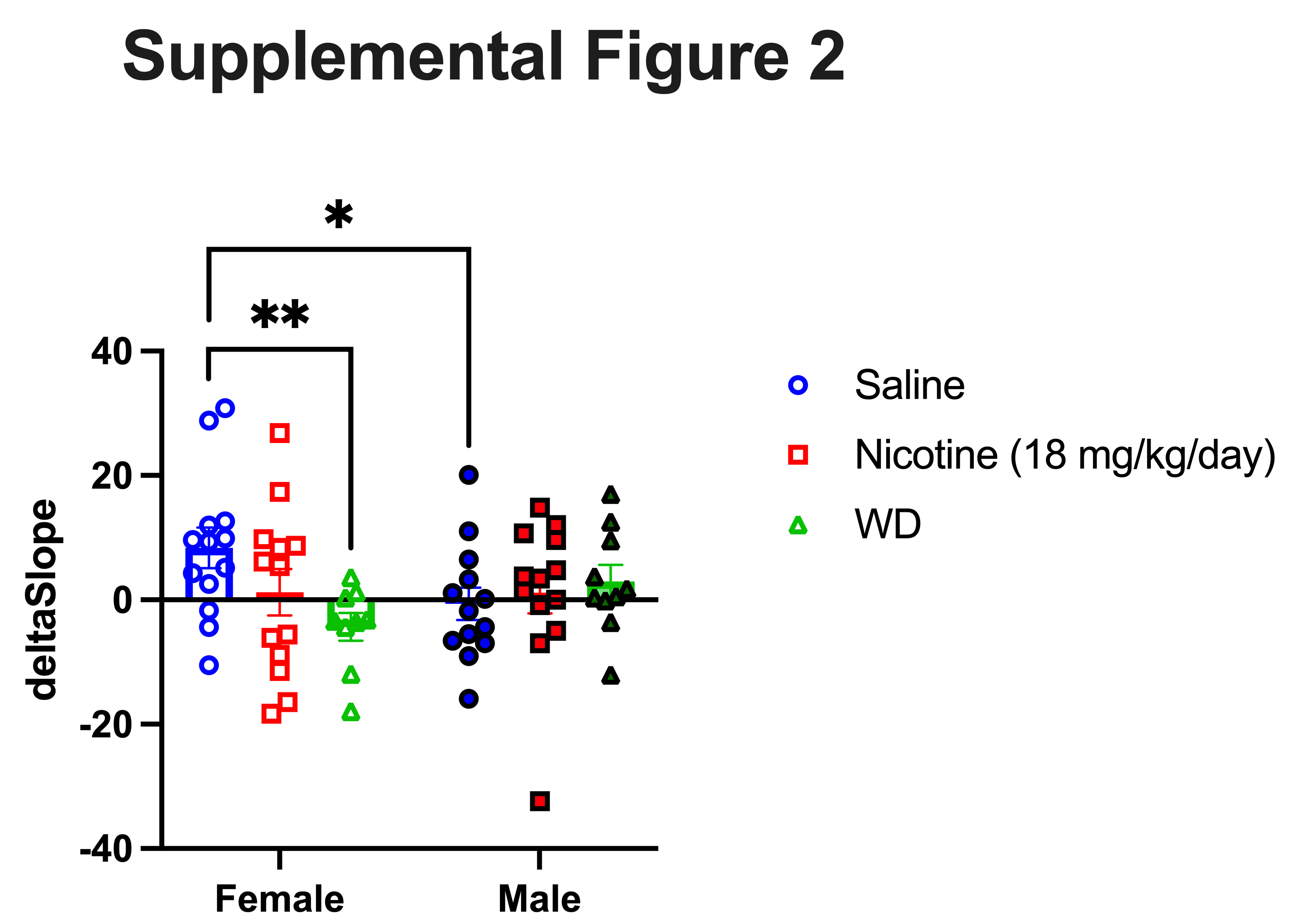

Supplement: Supplementary file 2 — Supplemental Figure 2: Impacts of Nicotine Treatment on Change in Extinction Rate During Fear Extinction. Bar graph show impact of sex and nicotine treatment on contextual fear extinction measured in change in slope. [n = 9 to 14 per treatment; error bars are SEM; * - P < 0.05, ** - P < 0.001] [file 13293_2024_656_MOESM2_ESM.tiff]

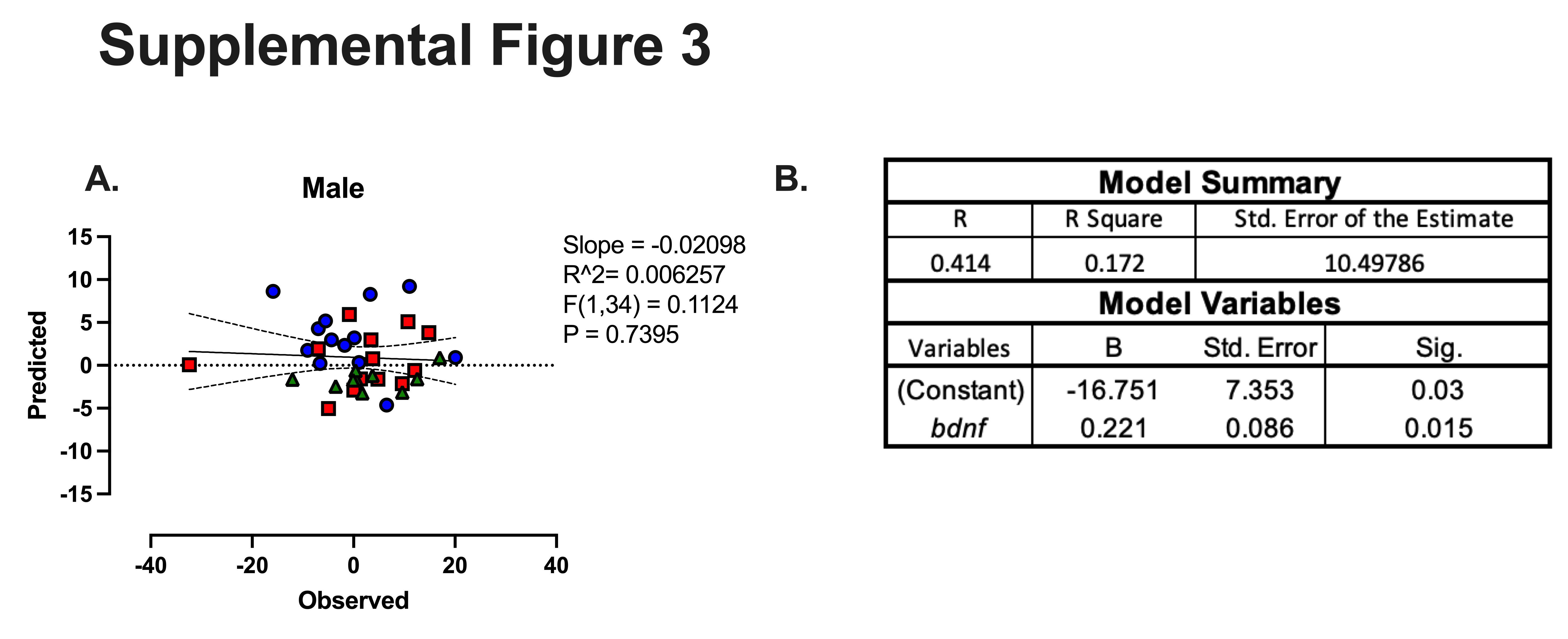

Supplement: Supplementary file 3 — Supplemental Figure 3: Modeling Delays in Fear Extinction of Male Mice Using Female Dorsal Hippocampal Gene Expression. (A) The graphed predicted versus observed values in change of slope for the male mice using the model generated using female dorsal hippocampal gene expression data. Predicted values were generated using female dorsal hippocampal gene expression values and observed change in slope values in SPSS statistics. Statistics presented on graph are the linear regression between predicted and observed change in slope values generated with GraphPad Prism with 95% CI. While the predicted values significantly correlated to the observed values of change in slope if females, it was not predictive when using male Dhip gene expression values. (B) The table are the coefficients used in the female gene expression model to predict changes in slope. bdnf, Brain Derived Neurotrophic Factor [n = 10 to 13 per treatment] [file 13293_2024_656_MOESM3_ESM.tiff]

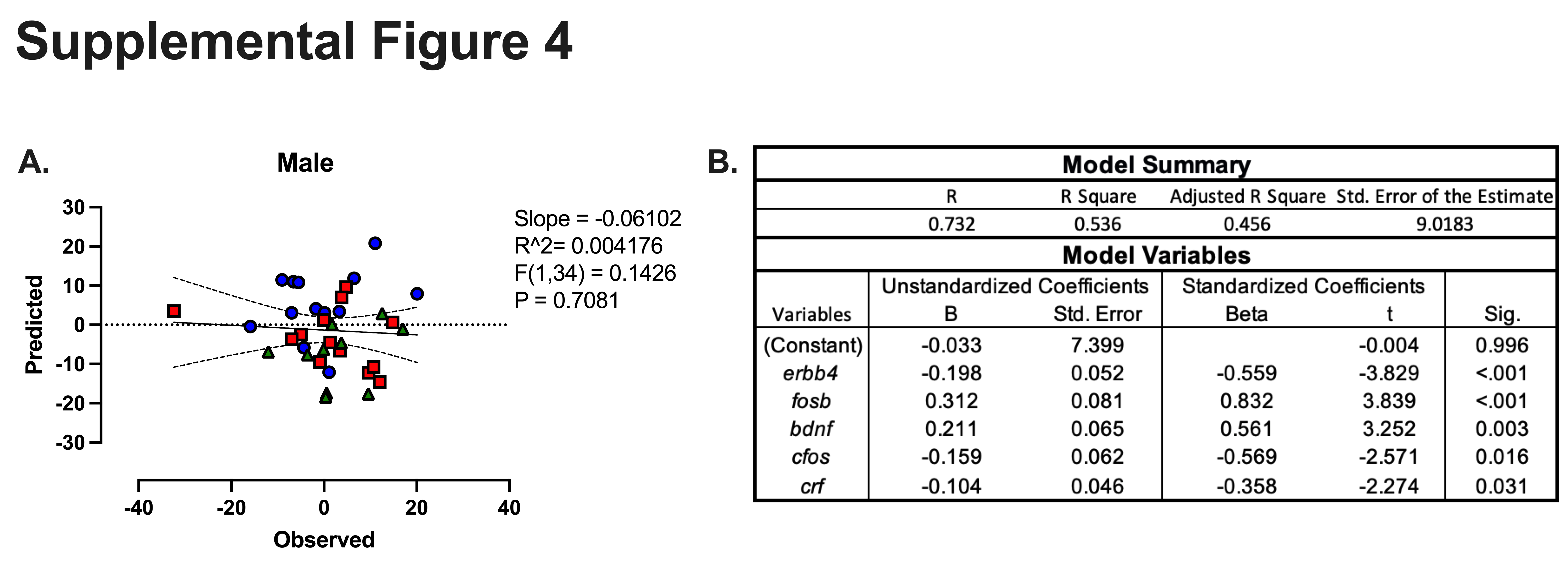

Supplement: Supplementary file 4 — Supplemental Figure 4: Modeling Delays in Fear Extinction of Male Mice Using Female Ventral Hippocampal Gene Expression. (A) The graphed predicted versus observed values in change of slope for the male mice using the model generated using female ventral hippocampal gene expression data. Predicted values were generated using female dorsal hippocampal gene expression values and observed change in slope values in SPSS statistics. Statistics presented on graph are the linear regression between predicted and observed change in slope values generated with GraphPad Prism with 95% CI. The model generated using female Vhip gene expression data was not predictive of change in slope in males. (B) The table are the coefficients used in our generated model to predict changes in slope. erbb4, Erb-B2 Receptor Tyrosine Kinase 4; fosb, FosB Proto-Oncogene, AP-1 Transcription Factor Subunit; bdnf, Brain Derived Neurotrophic Factor; cfos; Fos Proto-Oncogene, AP-1 Transcription Factor Subunit; crf, Corticotropin Releasing Hormone [file 13293_2024_656_MOESM4_ESM.tiff]

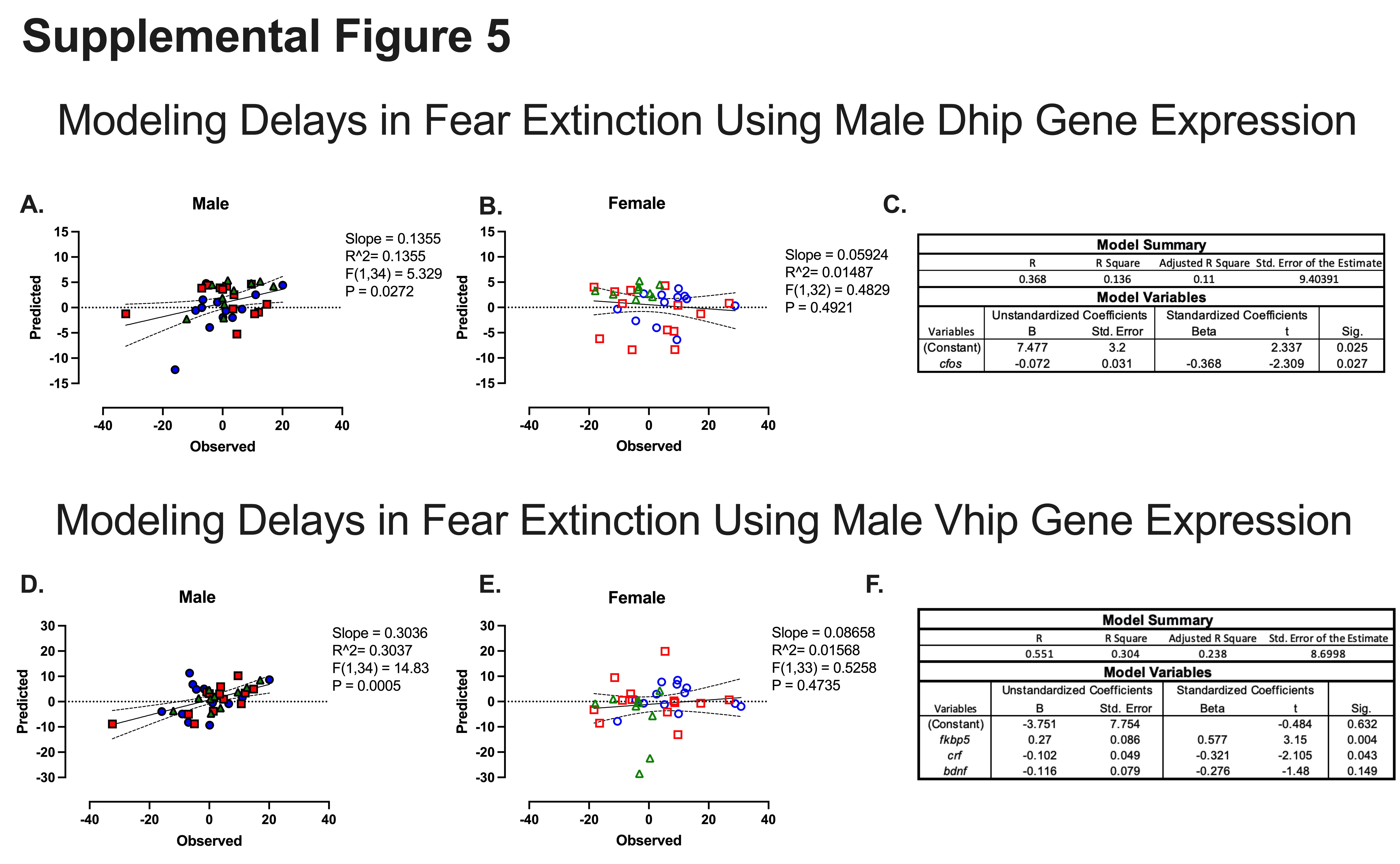

Supplement: Supplementary file 5 — Supplemental Figure 5: Modeling Delays in Fear Extinction Using Male Dorsal and Ventral Hippocampal Gene Expression. While we did not observe behavioral differences in treatment groups of the males, we performed the same linear regression using the male change in slope and all male Dhip (A-C) or all male Vhip (D-F) gene expression data to validate the same genes observed in females are not predictive in males with a different variable coefficient. (A) The graphed predicted versus observed values in change of slope for the male mice using the model generated using male dorsal hippocampal gene expression data. (B) The graphed predicted versus observed values in change of slope for the female mice using the model generated using male dorsal hippocampal gene expression data. Predicted values were generated using male dorsal hippocampal gene expression values and observed change in slope values in SPSS statistics. (C) The table are the coefficients used in our generated model to predict changes in slope using male dorsal hippocampal gene expression. cfos; Fos Proto-Oncogene, AP-1 Transcription Factor Subunit. (D) The graphed predicted versus observed values in change of slope for the male mice using the model generated from male ventral hippocampal gene expression data. (E) The graphed predicted versus observed values in change of slope for the female mice using the model generated from male ventral hippocampal gene expression data. Predicted values were generated using male ventral hippocampal gene expression values and observed change in slope values in SPSS statistics. (F) The table are the coefficients used in our generated model to predict changes in slope using male dorsal hippocampal gene expression. Fkbp5; fkbp5, FK506-Binding Protein 5; crf, Corticotropin Releasing Hormone; bdnf, Brain Derived Neurotrophic Factor; Transcription Factor Subunit. Statistics presented on graph are the linear regression between predicted and observed change in slope values generated [file 13293_2024_656_MOESM5_ESM.tiff]
